# Seroprevalence of RSV IgG Antibodies Across Age Groups in Poland After the COVID-19 Pandemic: Data from the 2023/2024 Epidemic Season

**DOI:** 10.3390/vaccines13070741

**Published:** 2025-07-09

**Authors:** Barbara Poniedziałek, Wiktoria Majewska, Katarzyna Kondratiuk, Aleksander Masny, Anna Poznańska, Karol Szymański, Katarzyna Łuniewska, Emilia Czajkowska, Bartosz Mańkowski, Lidia B. Brydak, Krzysztof Tomasiewicz, Robert Flisiak, Piotr Rzymski

**Affiliations:** 1 Department of Environmental Medicine, Poznan University of Medical Sciences, 60-806 Poznań, Poland; bpon@ump.edu.pl (B.P.); wiktoria.majewska@ump.edu.pl (W.M.); 2 Department of Virology, National Institute of Public Health NIH-National Research Institute, 00-791 Warsaw, Poland; ktomczuk@pzh.gov.pl (K.K.); amasny@pzh.gov.pl (A.M.); kszymanski@pzh.gov.pl (K.S.); kluniewska@pzh.gov.pl (K.Ł.); eczajkowska@pzh.gov.pl (E.C.); bmankowski@pzh.gov.pl (B.M.); lbrydak@pzh.gov.pl (L.B.B.); 3 Department of Population Health Monitoring and Analysis, National Institute of Public Health NIH—National Research Institute, 00-791 Warsaw, Poland; apoznanska@pzh.gov.pl; 4 Department of Infectious Diseases, Medical University of Lublin, 20-081 Lublin, Poland; tomaskdr@poczta.fm; 5 Department of Infectious Diseases and Hepatology, Medical University of Bialystok, 15-540 Białystok, Poland; robert.flisiak1@gmail.com

**Keywords:** respiratory syncytial virus, seroprevalence, epidemic season, infectious disease, respiratory infection

## Abstract

**Background/Objectives:** Respiratory syncytial virus (RSV) is a leading cause of respiratory infections across all age groups, with the greatest burden observed in young children and older adults. The COVID-19 pandemic significantly disrupted RSV circulation, resulting in an immunity gap and altered transmission dynamics. This study aimed to assess the seroprevalence of anti-RSV IgG antibodies in the Polish population during the 2023/2024 epidemic season. To our knowledge, this is the first study to characterize RSV seroprevalence at the population level in Poland. **Methods:** A total of 700 serum samples from individuals across different age groups were analyzed using a commercial assay to detect anti-RSV IgG antibodies. Seroprevalence and antibody levels, expressed as the index of positivity (IP), were examined by age and sex. **Results:** The overall seroprevalence of anti-RSV IgG antibodies was 91.4%. Antibody positivity increased markedly from 35.5% in infants aged 0–1 years to over 90% in children aged 4–5 years, reaching nearly universal levels in older age groups, including 99.1% in adults aged ≥60 years. Median IP values also rose with age, peaking in individuals aged ≥60 years. No significant differences in seroprevalence were observed between sexes, though older men showed slightly higher median IP values, potentially reflecting greater cumulative RSV exposure. **Conclusions:** This study provides key insights into the post-pandemic landscape of RSV immunity in Poland. The high seroprevalence across most age groups underscores widespread prior exposure, while the lower rates in infants highlight a continued vulnerability. These findings support the development and implementation of targeted immunization strategies, particularly for infants and older adults.

## 1. Introduction

Respiratory syncytial virus (RSV) is one of the most significant respiratory pathogens worldwide, commonly infecting and re-infecting individuals across all age groups [1]. However, the highest burden of severe disease is observed in children under five years of age and in the elderly, particularly those with underlying health conditions [2,3]. Globally, RSV is responsible for an estimated 33 million lower respiratory tract infections and more than 100,000 deaths annually among children under five [4]. In older adults, RSV-related morbidity and mortality are substantial, at times rivaling or similar to those attributed to nonpandemic influenza [5,6,7].

In Europe, initiatives like the RESCEU and PROMISE projects have significantly advanced the understanding of RSV epidemiology, generating country-specific burden estimates and modeling analyses. These studies highlight that RSV-associated hospitalizations in young children and older adults are a major public health concern, with Poland mirroring broader European trends of high hospitalization rates, particularly among infants under 12 months and adults over 60 years old [8,9,10]. The generated data revealed that RSV accounts for a substantial proportion of pediatric hospitalizations in Europe, with seasonal peaks typically occurring between October and February in temperate regions like Poland [10,11].

The COVID-19 pandemic profoundly disrupted RSV epidemiology. Sanitary restrictions and social distancing led to a near-absence of RSV circulation in the 2020/2021 epidemic season, followed by unseasonal rebounds in the 2021/2022 season [12,13,14]. A global multi-site analysis further demonstrated that respiratory viruses resurged asynchronously after the pandemic-related restrictions were lifted, with RSV re-emerging later than rhinovirus and endemic coronaviruses, suggesting virus-specific immunity dynamics [15]. Notably, the 2022/2023 season saw RSV epidemic peaks in the Northern Hemisphere occurring 1.9 months earlier than pre-pandemic baselines, with prolonged epidemic durations, underscoring persistent alterations in viral seasonality [16]. This surge has been attributed to an epidemiological compensatory phenomenon: the widespread implementation of public health measures during the COVID-19 pandemic significantly reduced RSV circulation, leading to a decline in natural exposure and immunity and, ultimately, a heightened population-level susceptibility once restrictions were lifted [17,18,19]. These shifts were not limited to RSV since similar trends were observed for influenza. In 2024, despite low reported incidence at the national surveillance level, a marked increase in influenza-related hospitalizations was recorded in Poland, particularly among vulnerable populations such as older adults and individuals with comorbidities [20]. This disconnect between community-level surveillance and clinical severity highlights the need for comprehensive monitoring of respiratory viruses, including RSV, in the post-pandemic context.

Amid these epidemiological shifts, 2023 marked a turning point in RSV prevention, with the approval of the first RSV vaccines targeting older adults and pregnant women, as well as a novel long-acting RSV-neutralizing monoclonal antibody for infants [21,22,23]. These medical advancements have opened new avenues for mitigating the impact of RSV, particularly in vulnerable populations [24].

Despite global interest, post-pandemic RSV seroprevalence data remain scarce in Central Europe, with no studies from Poland despite its documented hospitalization surge [8,11]. Previous studies primarily focused on pre-pandemic or early-pandemic periods [25,26,27,28], leaving a critical gap in understanding the current immune landscape following the unprecedented pandemic-related disruptions of 2020–2023. No such data exist for Poland, where the interplay of delayed RSV exposure, altered seasonality, and emerging immunization strategies necessitates updated serological insights.

Therefore, this study aimed to investigate the seroprevalence of IgG antibodies against respiratory syncytial virus in the Polish population across different age groups during the 2023/2024 epidemic season. By addressing this gap, the study offers critical insights into the post-pandemic immune status of the population, which helps refine national vaccination strategies, inform healthcare preparedness, and contribute to broader efforts in understanding RSV transmission dynamics in Central Europe.

## 2. Materials and Methods

### 2.1. Sample Collection

The study analyzed serum samples from 700 individuals collected between 1 October 2023 and 30 September 2024, at sixteen Voivodeship Sanitary and Epidemiological Stations (VSES) across Poland. These included stations in the following voivodeships: Lower Silesian, Kuyavian-Pomeranian, Lublin, Lubusz, Łódź, Lesser Poland, Masovian, Opole, Subcarpathian, Podlaskie, Pomeranian, Silesian, Holy Cross, Warmian-Masurian, Greater Poland, and West Pomeranian—ensuring comprehensive, country-wide geographic coverage. Sampling was conducted as part of the routine national screening of circulating antibodies against the hemagglutinin of influenza viruses. The sera were collected during routine diagnostic testing not related directly to respiratory viruses. These samples were anonymized and repurposed for serological surveillance in accordance with World Health Organization recommendations. Although this approach is practical and resource-efficient, it does not fully ensure random sampling and may not exclude selection bias. The information on the health status of the individuals was not available.

The sera were initially categorized into seven age groups (0–4, 5–9, 10–14, 15–25, 26–44, 45–64, and ≥65 years), with each group represented by 100 samples, in line with the influenza surveillance framework used in Poland and by the European Centre for Disease Prevention and Control [29]. For the purpose of this RSV-focused study, the samples were reclassified to better reflect RSV epidemiology and risk, resulting in the following age groups: 0–1 years (*n* = 45), 2–3 years (n = 33), 4–5 years (*n* = 43), 6–10 years (*n* = 89), 11–17 years (*n* = 132), 18–25 years (*n* = 58), 26–59 years (*n* = 188), and ≥60 years (*n* = 112). All sera were stored at −80 °C prior to testing and were inspected for hemolysis. None of the included children received monoclonal anti-RSV antibodies (i.e., nirsevimab or palivizumab), and none were born to mothers vaccinated against RSV. Additionally, none of the individuals eligible for RSV vaccination in the 2023/2024 season had received an RSV vaccine.

### 2.2. Determination of Anti-RSV IgG Antibodies

IgG antibodies specific to RSV were detected using a commercial enzyme immunoassay kit (EIA RSV IgG, TestLine Clinical Diagnostics, Brno, Czech Republic), following the manufacturer’s instructions. The assay is based on a sandwich enzyme immunoassay principle using native RSV antigens coated on microtiter plates. Serum samples were diluted 1:101 using the provided sample diluent (10 µL of sample + 1 mL of diluent), and 100 µL of each diluted sample was added to designated wells in duplicate. Positive control, negative control, and a cut-off control were included in each assay. The plate was incubated at 37 °C for 30 min, followed by five washing cycles with a 1:20 diluted wash buffer. Subsequently, 100 µL of horseradish peroxidase-conjugated anti-human IgG antibody was added to each well (excluding the blank), and the plate was incubated again at 37 °C for 30 min. After another wash step, 100 µL of tetramethylbenzidine substrate solution was added to each well and incubated at 37 °C for 30 min in the dark. The reaction was stopped by adding 100 µL of stop solution, and the optical density was measured at 450 nm using a microplate reader within 30 min of stopping the reaction. Results were evaluated by calculating the Index of Positivity (IP) as the ratio of sample absorbance to the mean absorbance of the cut-off control. According to cut-offs set by the manufacturer, samples with IP > 1.1 were considered positive for anti-RSV IgG antibodies. IP values among seropositive individuals were used to surrogate antibody levels.

### 2.3. Statistical Analyses

The data was statistically analyzed with Statistica v.13 (StatSoft, Tulsa, OK, USA). The continuous data did not follow a Gaussian distribution (checked with Shapiro–Wilk’s test). Fisher’s exact test and Mann–Whitney U test were employed to study sex differences in seroprevalence and IP, respectively. The association between IP and age was determined using Spearman’s correlation coefficient. A *p*-value below 0.05 was considered statistically significant.

## 3. Results

**Figure 1:**
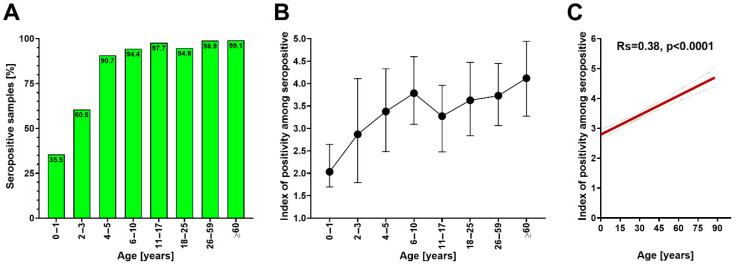
(**A**) Seroprevalence of anti-RSV IgG antibodies in the studied group (*n* = 700) stratified by age, (**B**) median (dot) and interquartile range (whiskers) values of the index of positivity of anti-RSV IgG antibodies across ages, and (**C**) correlation between age and index of positivity of anti-RSV IgG antibodies (presented as Spearman’s correlation coefficient with 95% confidence interval).

The seroprevalence of anti-RSV IgG antibodies in the entire group (*n* = 700) was 91.4% with a mean (±SD) IP value of 3.7 ± 1.3. It increased sharply from 35.5% in infants aged 0–1 years to over 90% in children aged 4–5 years, reaching near-universal levels (≥94%) in older age groups (Figure 1A). Among seropositive individuals, median IP values, a surrogate of antibody concentration, rose from 2.0 units in the youngest group to a peak of 4.1 in those aged ≥60 years (Figure 1B). The IP correlated positively with age in the studied cohort (Figure 1C).

No statistically significant sex-based differences in seroprevalence were observed in any age group (Table 1). Across most age groups, median IP values were similar between the sexes. The only marginally significant difference was found in adults ≥60 years, with men showing a higher median IP (Table 1).

## 4. Discussion

This study provides an essential snapshot of RSV immunity across lifespans in Poland at a critical juncture in respiratory virus epidemiology. The findings reveal when individuals are first exposed to RSV and the extent to which antibody levels accumulate over time due to repeated infections. By clarifying these patterns, the study supports more informed public health planning, tailored vaccination strategies, and better preparedness for seasonal RSV surges.

Our research demonstrated a clear age-dependent increase in both the prevalence and concentration of anti-RSV IgG antibodies. The low seroprevalence observed in infants aged 0–1 years (35.5%) reflects a lack of exposure to RSV through natural infection, but may also be associated with the waning of maternally derived antibodies, which are primarily transferred transplacentally during the third trimester and can offer partial, short-term protection in early infancy [30]. However, the levels of these IgG antibodies typically start to decline by 2 months of life, reaching seronegativity around 6 months [31]. Additionally, while both IgA and IgG antibodies can be present in breast milk, IgA is the dominant immunoglobulin class and plays a key role in mucosal immunity, rather than contributing to systemic IgG levels detectable by the serological assay employed in this study [32,33].

As passive maternal protection wanes and breast milk-derived antibodies may not contribute to systemic immunity, infants increasingly rely on their own immature immune systems to defend against RSV. This leaves infants susceptible to primary RSV infections, especially if one considers that infants up to 1 year have an altered, less effective immune response to RSV, marked by reduced activated regulatory T cells, decreased interleukin 33 production, and limited levels of chemokines that are known to limit viral replication, ultimately leading to poor inflammation control and antiviral action [34,35]. Such insufficient regulation is linked to the increased risk of more severe disease and is ultimately reflected in the highest rates of hospitalizations among children age groups, also in Poland [8]. This advocates for immunization strategies to decrease the RSV burden in infants. Real-world estimates show that maternal RSV vaccination is associated with high effectiveness against RSV-associated lower respiratory tract disease, including severe forms leading to hospitalization from birth to 3 months and sustained to the age of 6 months [36]. Similarly, a single dose of nirsevimab in infants effectively reduced pediatric emergency department visits related to RSV-associated bronchiolitis and subsequent hospitalizations [37].

The sharp rise in seroprevalence to 60.6% in the 2–3-year age group and to over 90% in children aged 4–5 years indicates that most children encounter RSV early in life. This pattern is consistent with studies from various other regions, including Thailand and the Netherlands, where nearly all children are seropositive by the age of 3 years [26,27,38,39]. Our work shows that such a high seroprevalence remains in older children and all adult age groups.

Among seropositive individuals, antibody concentrations increased with age, from a mean PI value of 2.0 in infants to 4.1 in those aged ≥60 years. This gradual rise suggests cumulative RSV exposures over the lifetime. The serosurveys conducted in other countries, such as the United States, India, Italy, or Germany, show that 85–95% of adults aged ≥60 reveal antibodies against the fusion protein of RSV [28,40,41,42]. However, increased IgG levels do not confer long-term immunity. Studies have shown that natural RSV infection induces only partial and short-lived protection, allowing for frequent reinfections throughout life. For instance, research indicates that within just over two years of a natural RSV infection, the majority of adults experienced multiple reinfections, with almost half even three or more, despite having high levels of neutralizing antibodies [43]. Similarly, in early childhood, at least one-third of children who had undergone an RSV infection were susceptible to reinfection in subsequent years, suggesting that primary RSV infection provides only partial protection against reinfection [44]. One important factor contributing to this insufficient immunity is the nature of RSV antigen presentation. The prefusion conformation of the F protein, which elicits the most potent neutralizing antibodies, is structurally unstable and often transitions into the postfusion form before it is processed by antigen-presenting cells. This results in a biased immune response focused on non-neutralizing epitopes of the postfusion protein [45]. Consequently, repeated natural infections reinforce this skewed immunological memory by driving clonal expansion of B cells that target accessible but poorly neutralizing regions. This phenomenon leads to high serum titers of IgG antibodies that are functionally weak. In line with this, one study has shown that older adults who had not experienced RSV infection in a given season had four-fold higher preseason neutralizing titers compared to those who had been infected, highlighting the immune-dampening effect of repeated, non-protective RSV exposures [46]. These findings underscore the need for effective vaccination strategies, as natural infection does not lead to durable immunity against RSV [47].

Therefore, despite the high seroprevalence of anti-RSV IgG antibodies in individuals aged ≥60 years (99% in the present study) and increased levels, older adults remain at significant risk for severe lower respiratory tract infections, hospitalization, long-term complications, and mortality due to RSV. This vulnerability persists despite the high prevalence of anti-RSV IgG antibodies, suggesting that these antibodies alone may be insufficient for protection. A study analyzing data from 12 US states found that among adults aged ≥60 years hospitalized with laboratory-confirmed RSV infection, 17.0% were admitted to an intensive care unit, 4.8% required mechanical ventilation, and 4.7% died [48]. Factors such as immunosenescence, i.e., the gradual deterioration of the immune system associated with aging, can impair the quality and functionality of the innate immune response and adaptive parameters, including the efficacy of antibodies [49]. Consequently, although older adults may have high levels of anti-RSV IgG, as shown in the present study, they may not provide adequate protection against infection or severe disease. This is consistent with findings from seroprevalence surveys that also assessed neutralization capacity, which show that despite nearly universal anti-RSV IgG seropositivity in older individuals, the serum of the majority lacks any neutralizing activity against the virus [25,42]. This functional deficiency leaves them vulnerable not only to infection but also to more severe clinical outcomes, especially in the presence of other risk factors such as an immune system aging and underlying chronic conditions (e.g., chronic obstructive pulmonary disease, asthma, coronary artery disease, diabetes mellitus) [49,50]. In turn, clinical and real-world evidence demonstrates that RSV vaccines are effective in preventing RSV-associated hospitalizations in adults aged 60 and older and can reduce the overall burden of RSV-related morbidity and mortality in this vulnerable population [22,23,51,52,53].

Importantly, when stratified by sex, we found no significant differences in seroprevalence between males and females across all age bands. However, one should note that numerically, in children aged 2–3 years, the seroprevalence in males was 24% higher. In fact, there is some evidence that boys may be more susceptible to RSV infection, its more severe course, hospitalization risk, and odds of RSV-triggered asthma [54,55,56]. IP values, a surrogate of antibody levels, were also mostly similar between sexes, except in adults aged ≥60 years, with men displaying slightly higher median IgG indices than women. This mirrors findings from other cohorts where sex did not consistently influence RSV-specific IgG in children or adults, although a few studies have noted modestly elevated titers in adult males, e.g., a Minnesota adult study reported higher RSV IgG in men [40]. Whether this difference confers enhanced protection in older men remains unclear since functional characteristics such as neutralizing capacity and T-cell-mediated immunity may play a larger role in clinical outcomes. Instead, it could reflect a higher cumulative exposure to RSV over the lifetime, possibly due to behavioral, occupational, or biological factors.

The present study was conducted during the 2023/24 epidemic season, following the discontinuation of COVID-19 as a Public Health Emergency of International Concern in 2023 and after various sanitary restrictions were already lifted in many European countries in 2022 [57]. Therefore, notable RSV resurgences were already observed in the 2022/2023 season, with increased case counts and altered seasonality reported in multiple countries. These earlier post-pandemic resurgences and increased RSV activity during the 2023/2024 season may have also contributed to the elevated seroprevalence detected in our study, particularly among older children and adults, reaching nearly universal levels.

Notably, in our study, none of the included children received monoclonal anti-RSV antibodies, i.e., nirsevimab or palivizumab, and none were born to mothers vaccinated against RSV. Furthermore, none of the individuals eligible for RSV vaccination in the 2023/2024 season received the RSV vaccine. These facts are important to consider when interpreting the observed seroprevalence, as they indicate the exposure to natural infections, not confounded by immunization.

This study provides the first post-pandemic seroepidemiological data on anti-RSV IgG in the Polish population, offering important insights into immunity across age groups in a large, demographically diverse cohort, which is a strength of our investigation. The use of a commercial assay and robust stratification by age and sex strengthens the reliability of the findings. However, the study has limitations. First, the cross-sectional design prevents the assessment of seasonality in antibody dynamics. Second, although sera were collected by all VSES in Poland, ensuring country-wide coverage, they were collected during routine clinical testing, which may not fully ensure random sampling and may not exclude selection bias. Third, the assay measured total IgG levels, which do not directly reflect functional neutralization or protection. Indeed, the presence of anti-RSV IgG does not necessarily equate to protective immunity, particularly in vulnerable populations such as older adults [42]. Fourth, potential confounders such as comorbidities and a history of previous RSV infections or exposures were unavailable. Fifth, in the infant group, the assay could not distinguish between maternally derived IgG antibodies and those resulting from early postnatal infection, which limits the interpretation of the origin of seropositivity in this age category.

## 5. Conclusions

This research demonstrates a high seroprevalence of RSV-specific IgG antibodies in the Polish population, with a clear age-dependent increase in both prevalence and antibody levels. Despite nearly universal seroprevalence, older adults remain at risk for severe RSV-related disease. Therefore, an increased anti-RSV IgG seropositivity and concentrations in the elderly reflect cumulative exposure to the pathogen and should not be interpreted as a marker of enhanced immunity. High seroprevalence not equaling high or durable protective levels underscores one of the essential paradoxes in the RSV-related adaptive response, arising from the complex nature of immunity against respiratory viruses like RSV. The presented data reinforce the critical role of maternal and early childhood immunization efforts, as early-life immunity gaps can have lasting consequences on the population health burden. They also call attention to the necessity of addressing immunosenescence and other age-related immune system changes through tailored vaccine formulations and booster policies. In summary, the insights of the present study should inform national immunization programs in Poland and guide public health planning for RSV prevention in the post-pandemic era.

## Figures and Tables

**Table 1:** Association between seroprevalence and index of positivity (median and interquartile range of anti-RSV IgG antibodies in the studied cohort (*n* = 700) in relation to sex.

Age Group	*n*	Seroprevalence [%]		Index of Positivity Among Seropositive	
		Men	Women	Men	Women
**0–1**	45	36	37	2.4 (1.7–2.6)	2.0 (1.7–2.4)
		*p* = 0.60		*p* = 0.96	
**2–3**	33	70	55	3.2 (2.1–4.3)	2.1 (1.3–3.1)
		*p* = 0.16		*p* = 0.15	
**4–5**	43	89	94	2.9 (3.2–3.8)	3.4 (2.5–4.4)
		*p* = 0.57		*p* = 0.36	
**6–10**	89	89	97	3.5 (2.7–4.4)	3.7 (3.1–4.2)
		*p* = 0.27		*p* = 0.69	
**11–17**	132	98	98	3.4 (2.4–4.2)	3.2 (2.5–4.0)
		*p* = 71		*p* = 0.77	
**18–25**	58	97	93	3.6 (2.7–4.3)	3.6 (2.9–5.0)
		*p* = 0.45		*p* = 0.23	
**26–59**	188	100	98	3.9 (3.4–4.6)	3.6 (2.8–4.4)
		*p* = 0.34		*p* = 0.09	
**≥60**	112	98	100	4.5 (3.6–5.0)	4.0 (3.1–4.6)
		*p* = 0.49		*p* = 0.04	

## Data Availability

The data that support the findings of this study are available from the corresponding author upon reasonable request.
